# Statistical and regulatory considerations in assessments of interchangeability of biological drug products

**DOI:** 10.1007/s10198-014-0589-1

**Published:** 2014-05-16

**Authors:** Lászlo Tóthfalusi, László Endrényi, Shein-Chung Chow

**Affiliations:** 1 Department of Pharmacodynamics, Semmelweis University, POB 370, Budapest, 1445 Hungary; 2 University of Toronto, Toronto, ON M5S 1A8 Canada; 3 Duke University, Durham, NC 27710 USA

**Keywords:** Bioequivalence, Equivalence tests, Follow-on biologics, Biosimilars, Interchangeability, I1, Y8

## Abstract

When the patent of a brand-name, marketed drug expires, new, generic products are usually offered. Small-molecule generic and originator drug products are expected to be chemically identical. Their pharmaceutical similarity can be typically assessed by simple regulatory criteria such as the expectation that the 90 % confidence interval for the ratio of geometric means of some pharmacokinetic parameters be between 0.80 and 1.25. When such criteria are satisfied, the drug products are generally considered to exhibit therapeutic equivalence. They are then usually interchanged freely within individual patients. Biological drugs are complex proteins, for instance, because of their large size, intricate structure, sensitivity to environmental conditions, difficult manufacturing procedures, and the possibility of immunogenicity. Generic and brand-name biologic products can be expected to show only similarity but not identity in their various features and clinical effects. Consequently, the determination of biosimilarity is also a complicated process which involves assessment of the totality of the evidence for the close similarity of the two products. Moreover, even when biosimilarity has been established, it may not be assumed that the two biosimilar products can be automatically substituted by pharmacists. This generally requires additional, careful considerations. Without declaring interchangeability, a new product could be prescribed, i.e. it is prescribable. However, two products can be automatically substituted only if they are interchangeable. Interchangeability is a statistical term and it means that products can be used in any order in the same patient without considering the treatment history. The concepts of interchangeability and prescribability have been widely discussed in the past but only in relation to small molecule generics. In this paper we apply these concepts to biosimilars and we discuss: definitions of prescribability and interchangeability and their statistical implementation; the relation between bioequivalence and interchangeability for small-molecule drug products; regulatory requirements and expectations of biosimilar products in various jurisdictions; possible statistical approaches to establish the similarity and interchangeability of biologic drug products; definition of other technical terms such as switchability and automatic substitution. The paper will be concluded with a discussion of the anticipated future use of interchangeability of biological drug products.

## Introduction

New drug development is a lengthy, expensive, and complex process. The high and increasing cost of the development is reflected in the increasing price which is a constant source of concern for governments, health care providers and generally for the public [1]. However, when the patents protecting a drug product expire, then competitor products can appear on the market. A competitor’s drug products are called generics. Generics contain the same active ingredient in the same amount as the originator formulation but at a lower price [2].

It is a fundamental principle in clinical pharmacology that the therapeutic effect is solely determined by the plasma concentration of the active molecule. Consequently, regardless of the formulation factors, if the plasma concentrations of two products are the same or very similar then the therapeutic effects also must be practically the same. This is the basic concept for the regulatory approval of generic products.

Biological drugs are not synthesized chemically but are made by living organisms. They are much larger than small-molecule drugs and have much more complicated, primary, secondary, tertiary and quaternary structures. They may exhibit immunogenicity (unwanted immune response) when the immune system recognizes a large molecule as a foreign invader. Their manufacturing procedures are difficult and sensitive to environmental conditions such as temperature, light and pressure. Consequently, a “generic” biological product cannot be identical but only similar (“biosimilar”) to the drug of an originator. The two products should be highly similar so that their clinical effects should also be very close. The issue of what is “highly similar” is a matter of judgment and is based on the totality of all the evidence. This involves several considerations including structural, functional, analytical, immunological, pharmacokinetic, pharmacodynamic, manufacturing and clinical similarities.

Even if two products are judged and declared to be biosimilar, they may not be readily substituted automatically within individuals from one to the other by pharmacists. This kind of interchangeability requires much more convincing evidence, which can be obtained only from additional investigations and calls from further, careful judgment.

This article discusses statistical and regulatory aspects of the interchangeability of biologicals. First, definitions of interchangeability and conditions for its application will be presented. This will be followed by past experience for the evaluation of bioequivalence and interchangeability for small-molecule drug products. Regulatory requirements and expectations for biosimilar products in various jurisdictions will then be discussed. Thereafter, statistical approaches to demonstrate the similarity and interchangeability of biological drug products will be summarized. Finally, the prospective future use of the interchangeability of biologics will be contemplated.

The first part of this paper summarizes the developments in this field in the last 20 years. The statistical aspects of bioequivalence are a constantly evolving area. There are many facets of this problem, but in this communication we discuss only the statistical and regulatory aspects. Finally, we shall summarize how these statistical principles are reflected in the legislation in different jurisdictions.

## Average bioequivalence for the approval of generic products

Bioequivalence studies are required for granting marketing authorization for generic medicinal products. The exact requirements slightly differ between main jurisdictions such as the US, EU, and Japan, but the general approach is the same.

The approval is based on the results of a bioequivalence study. The plasma concentrations of the generic (called test product, T) and the originator (reference, R) formulations are compared in a limited number (somewhere between 18 and 60) healthy volunteers in a crossover fashion. Half of the volunteers are randomly assigned to get the test drug and after it is eliminated from the body, they receive the reference product. For the other half of the group the order is reversed, receiving first the reference and then the test product. After drug ingestion, several blood samples are obtained and the concentrations of the active substance are determined.

To demonstrate bioequivalence, it should be shown that derived parameters from the concentrations, like the areas under the curve (AUC) of the test and reference products, are not really different, they are similar to each other.

The currently used method to assess bioequivalence was proposed by the FDA’s statistician Schuirmann [3] and is called the “two one-sided tests” (TOST) method. In the first step, the logarithms of the relevant pharmacokinetic parameters are taken. In a second step, after the eliminating period and sequence effects, differences between individual values are calculated. We denote the mean of individual differences by m_T_−m_R_.

In the third step, two one-sided t-tests are performed:

1 $$ {\text{Test 1}}\quad m_{\text{T}} - m_{\text{R}} < - \Theta $$

2 $$ \text{Test} \, 2\quad m_{\text{T}} - m_{\text{R}} > \Theta $$

If both tests are rejected at the 0.05 level, then we have established, with 90 % confidence, that the mean of the individual logarithmic differences is between −Θ and Θ. Θ is a regulatory cutoff, usually log (1.25). It is common that the TOST result is interpreted after back-transformation, that is, after taking the antilogarithms of Eqs. 1 and 2. If both equations are rejected then it can be stated, with 90 % confidence, that the geometric mean of the individual ratios is between 0.80 and 1.25. If this holds, then a generic product is approvable at least from the bioequivalence viewpoint.

The TOST procedure assesses if the difference between the (logarithmic) averages satisfies the regulatory criterion for bioequivalence (Eqs. 1, 2). Therefore, it is a test for determination of average bioequivalence (ABE).

## Critique of the average bioequivalence concept

Testing bioequivalence in the way presented above has become the general, standard procedure. However, it can be subject to questions and criticisms. There are at least three issues:

- First, ABE focuses only on the differences between the mean values and neglects the possible differences between the variances. It is possible that due to technological problems one of the products will not have constant quality, and due to the fluctuations in critical technological parameters, a patient sometimes will be overdosed and sometimes will be underdosed. Quality differences between two drug products could be measured by comparing the between- and within-subject differences, but the current ABE decision criterion is independent of this parameter.
- The second problem is what is called subject-by-formulation interaction. In theory it is possible that there are subjects who have the highest value with the test formulation but have the lowest value with the reference product. Also, vice versa, other subjects with the lowest value with the reference formulation can have the highest value with the test product. The means and even the variations of test and reference formulations could be the same, nevertheless, individuals would exhibit differing effects after receiving the two drug products.
- The third issue is related to the fact that the maximum allowed difference between the means, (the regulatory cutoff) is fixed, regardless of the between- or within-subject variances. It could be argued that, if a drug has a narrow therapeutic window, the applied regulatory cutoff allows too wide difference between the means of the products. In other cases, the regulatory cutoff is too strict if, for biological reasons, the drug concentration in the plasma fluctuates very widely. For such drugs, called highly variable drugs, bioequivalence can be demonstrated only if an unreasonably high number of volunteers, a hundred or even more, participate in the bioequivalence study. The associated development costs could be a deterring factor for most generic developers and would wipe out the economic advantage of the generic alternative.

## Prescribability and interchangeability

Anderson and Hauck recognized [4] the deficiencies of the standard method (Eqs. 1, 2) for assessing bioequivalence. In their original publication [4] Anderson and Hauck focused only on generic drugs, as in 1990 the term ‘biosimilarity’ and the category of biosimilar drugs as a whole was unknown.

To start with, they distinguished between two conditions in which a patient could encounter a generic product. The first scenario is that the patient had no prior exposure to a drug in any of its forms, i.e. if s/he is naïve to the drug. In such situations, any of the approved products are *prescribable* provided that their safety and efficacy are satisfactory and, therefore, they have been approved by regulators. However, after the patient has started to receive one of the products, it may not be substituted by another formulation.

The second scenario is when the patient is already taking the drug but opts for a cheaper alternative for economic reasons. In this scenario the different products must be therapeutically equivalent. Therefore, the products must be *switchable* within individuals. The switch usually occurs from the reference product to the test preparation (R → T) but alternative directions are also possible like the T → R and T → T′ switch; here T′ is a second generic, test product. The products R, T and T′ must be interchangeable in any possible sense to achieve the same therapeutic effect after a switch.

Depending on the condition, two test models were proposed. To describe prescribability quantitatively the so-called population bioequivalence (PBE) model was developed. This required comparison of the population distributions of the relevant pharmacokinetic parameters. A simple parallel-group design study was considered adequate to establish PBE.

In contrast, interchangeability could be demonstrated by satisfying the requirements of a model for individual bioequivalence (IBE). Essentially, IBE is an extended version of ABE and it eliminates the shortcomings of ABE by adding new terms to the statistical decision rule. But the implementation of IBE calls for a more complex study design: IBE requires investigations with a crossover design in which both drug products are measured (at least) twice within individuals.

Individual BE and interchangeability for small-molecule drug products were widely discussed for about a decade, starting in the early 1990s. However, the need for more complicated studies, three or even more than three period crossover studies was questioned. The draft guideline released by the FDA [5] met serious criticism and was later superseded by another guideline [6] in which the FDA reverted back to ABE. The concept of IBE was acknowledged but was never accepted by the CHMP, the European regulatory body.

## Biosimilars, switchability and interchangeability

A statement of *interchangeability* between a generic *biologic* and a reference product is distinct from assessments of their *biosimilarity*. Considerations in various jurisdictions will be discussed below.

### United States

The Biologics Price Competition and Innovation (BPCI) Act of 2009 in the US, clearly defines, separately, both biosimilarity and interchangeability [7].

The BPCI Act defines the term biosimilar (or biosimilarity) as when the biological product is *highly similar* to the reference product (notwithstanding minor differences in clinically inactive components), and also when there are *no clinically meaningful differences* between the biological product and the reference product in terms of safety, purity and potency.

The BPCI Act of the US provides explicit definitions and conditions for interchangeability. “The terms *interchangeable* and *interchangeability* mean that: (1) the biological product is *biosimilar* to the reference product; (2) the biological product can be *expected* to produce the *same clinical result* as the reference product *in any given patient*; (3) for a product administered more than once, the *risks of safety and reduced efficacy of alternating and switching* are not greater than with the use of the reference product without alternating or switching.” (Emphasis has been added).

There is a clear and strong distinction between *biosimilarity* and *interchangeability*. Biosimilarity is a precondition of interchangeability. The regulatory approval of the biosimilarity of two products does not imply at all their interchangeability. If two biologic products have already been approved and are marketed, an additional application must be submitted which would demonstrate that conditions for their interchangeability are also satisfied.

The strong distinction and hierarchy between the biosimilarity and interchangeability of biologic products is in sharp contrast to the close parallel between the bioequivalence and interchangeability of small-molecule drug products.

However, while the BPCI Act distinguished between biosimilarity and interchangeability, such a distinction is still to be seen in the corresponding FDA guidelines. This has led to legal controversies.

### Automatic substitution in the United States

The BPCI Act of the US also decrees important consequences of the approval of interchangeability between biologic drug products. The BPCI Act states that the interchangeable “biological product may be substituted for the reference product without the intervention of the health care provider who prescribed the reference product” [7]. Consequently, according to the BPCI Act, if a test product is judged to be interchangeable with the reference product then it may be substituted, even alternated, without a possible intervention, or even notification, of the prescribing physician.

This is a very permissive stipulation and has raised concerns at the level of state legislations. The basis of these concerns is that substitution of a drug product by another could give rise to changes of the safety and efficacy in individuals. It is expected, however, that risks associated with these changes are well controlled.

For instance, when a patient is switched from a reference formulation to a test product, R → T, then the associated risk should not be higher than when the subject is exposed to the reference product twice: R → R′. This was the sense of earlier considerations of individual BE. Scenarios of switching and alternating have not been considered by the FDA. On the other hand, until end of 2013, ten states introduced measures which permitted the substitution of biological products. Thereby, they contravened the federal BPCI Act which had required a separate, additional designation of interchangeability. The decrees of the states also prohibited automatic substitution by pharmacists. Four others approved such measures, though three put sunset clauses on the notification requirement, meaning the restriction will die after a certain number of years [8].

Thus it seems that a regulation which does not answer possible concerns may achieve an outcome which could be opposite from its intended purpose. Biologics are complicated drugs and their clinical responses are usually also complicated and sensitive to various conditions. Consequently, prescribing physicians may want to monitor the responses following the switching of drug products. If the consequence of approved interchangeability were free, relaxed substitution, then physicians, and patients, may prefer not to use the option of interchangeability. But the legal situation is in constant flux. For example, on 12 October 2013, California Governor Jerry Brown vetoed legislation known as SB 598, the legislation which would have forbidden a pharmacist from substituting a biosimilar for a brand-name biological if a physician ticked a ‘do not substitute’ box [9].

### Canada

In Canada, the term “substitutability” is applied to two products which can both be used in lieu of the other during and within the same treatment period, i.e. for interchangeability as discussed in this paper. The position of Health Canada on the substitution of biological products (biosimilars are called “subsequent-entry” products in Canada) is clear but rather soft: “Health Canada does not support the automatic substitution of a subsequent-entry biologic for its reference biologic drug. Health Canada therefore recommends that physicians make only well-informed decisions regarding therapeutic interchange” [10]. The position of Health Canada does not have legal authority. Funds for the reimbursement of pharmaceutical expenses are dispensed by the provinces. They will be keen to promote introduction of new generic biologic products, i.e. their prescribability. The interchangeability of biologic products, and the related conditions, will be diverse and controversial.

### European Union

The EU was the first region in the world to have set up legal frameworks and regulatory pathways for biosimilars. In fact, the word “biosimilars” was coined during this legislative process, and legal terms for “similar biologicals” are different in other jurisdictions. They are called follow-on biologics (FOB) in the US, subsequent-entry biologics (SEB) in Canada, and follow-on proteins (FOP) in Japan. In this paper we use biosimilars as shorthand for similar biologicals regardless of geographical implications, because the concepts behind these names are the same though local variations exist. The concept of a “similar biological medicinal product” was adopted in EU pharmaceutical legislation in 2004 and came into effect in 2005. The European Medicines Agency (EMA) was the first to lay down an abbreviated regulatory pathway for biosimilars. As of December 2013, 17 biosimilars within the product classes of human growth hormone, granulocyte colony-stimulating factor and erythropoietin TNF-alpha have been approved in Europe [11]. Only the EMA can grant marketing authorization for biosimilars in the European Union. More precisely, the European Commission issues the decisions concerning the authorization of these medicinal products on the basis of the scientific opinions from the EMA. The resulting marketing authorization is valid in all EU Member States.

For granting approval for a biosimilar product, EMA requires clinical trials, including comparability studies to the originator product, to demonstrate safety and efficacy. Unlike generics, demonstration of bioequivalence is not enough for biosimilars. Additionally, the prospective market authorization holder also must demonstrate lack or at least comparable immunogenicity in long-term clinical trials. Guidelines were established to provide further details on specific needs for demonstrating biosimilarity for four primary product classes.

The EMA has made it clear that a biosimilar is not the same as a generic drug, and has handed the interchangeability issue over to individual countries. As an EMA document states [12]: “The EMA evaluates biosimilar medicines for authorization purposes. The Agency’s evaluations do not include recommendations on whether a biosimilar should be used interchangeably with its reference medicine. For questions related to switching from one biological medicine to another, patients should speak to their doctor or pharmacist.” Some countries went further and legislation has been passed which prohibits automatic substitution by pharmacists [13]. Given that most currently approved biosimilars are applicable only in hospital settings, the impact of such a strict prohibition is moderate. Much more important is the reimbursement policy [14] where there are many national variations. One of the options is that new patients need to be treated with a biosimilar product if it is available, otherwise the treatment is not reimbursed. However, patients who have already been treated with a brand-name biological will continue receiving the same biological brand. Consequently, such a policy allows prescribability but not the interchangeability of biosimilar products. To describe these different scenarios, a European Consensus Group on Biosimilars proposed to make distinctions between interchangeability, switching and substitution. According to the consensus document [15]:

- Interchangeability means changing one medicine with the agreement of the prescriber.
- Switching means a decision by the treating physician to exchange one medicine for another.
- Substitution means of the practice of dispensing one medicine instead of another equivalent and interchangeable medicine at the pharmacy level without consulting the prescriber.

Making such distinctions between the post-approval use of biosimilars might be needed to describe the different possibilities. But reloading the meaning of these common English words which so far have been used as synonyms, or with different meaning in the scientific literature, could lead to confusion. The word switching appears to be a particularly bad choice of wording because native English speakers seem to associate switching with the inadvertent exchange of medicines.

### Statistical considerations on the interchangeability of biological products

The definitions of the American BPCI Act for the interchangeability of biologicals were outlined earlier. One of them is particularly forbidding, apparently categorical: “the biological product can be *expected* to produce the *same* clinical result as the reference product in *any* given patient”. Responses of patients are variable and the same clinical response may not be anticipated. Furthermore, the expectation of the *same* result in *any* given patient could be interpreted to mean that if an individual experiences an unexpected (not to say, not the “same”) therapeutic or adverse effect s/he may well seek recourse. Lawyers, notably in the US, would undoubtedly encourage this.

However, the word “*expected*” in the above phrase could be interpreted that the “same clinical result in any given patient” is not a categorical rule but only an expectation. “Expectation” also has a probabilistic meaning which involves some stated statistical assurance. It is hoped that this interpretation will be duly considered and adopted.

While many biologicals have long terminal half-lives, some don’t. With these, it is possible to contemplate crossover investigations which would enable the assessment of features of interchangeability. Notably, switching between two biological products, either reference (R) or test (T), such as R → T, T → R, R → R′, T → T′, could be investigated by Balaam’s 4 × 2 crossover design with 4 sequences and 2 periods [16, 19], including RT, TR, RR′, TT′. Here, e.g., RT refers to a sequence in which first the reference and then, after a sufficient washout period, the test product is provided to each individual. R and R′ denote two administrations of the reference product. Similarly, alternating between the test and reference products, i.e. R → T→R′, T → R→T′, could be studied by a 2-sequence, 3-period design with the sequences of RTR′, TRT′. Finally, both switching and alternating could be investigated by modifying Balaam’s design in four sequences and either two or three periods: TT′, RR′, TRT′, RTR′.

Little work has been undertaken so far on statistical procedures for the assessment of interchangeability of biologicals. Chow et al. [17] developed switching and alternating indices. These were based on the concept of a biosimilarity index which had been defined earlier [18]. This evaluates whether the probability of concluding average biosimilarity (corresponding to ABE) in a contrast of the biosimilar test and the reference products (T vs R) is similar to the “reproducibility” probability obtained in a comparison of two applications of the reference product (R vs R). The switching and alternating indices extend this concept to the more complicated designs outlined above.

Statistical approaches for evaluating the interchangeability of biological products will be undoubtedly developed further in the near future. They will provide a useful tool for the regulatory assessment of the evidence.

## Conclusions

The approach to the interchangeability of drug products is very different for small-molecule drugs compared with biologicals. With small-molecule drugs, approval automatically indicates, in most cases, interchangeability. Generic competition is based on that principle, though this fact is rarely recognized. In contrast, regulatory approvals of biosimilar products neither implicate nor prohibit interchangeability. Drug regulatory authorities are very cautious to make any statement in this regard.

In the US, the BPCI Act theoretically allows, but places very strong limitations on, substitutions of biologics. More importantly, the practical details of how to demonstrate interchangeability are lacking. Theoretically, the individual BE concept could serve as a theoretical framework to assess interchangeability, but there are other proposals [19]. None of them have been accepted yet by US regulators, but strong differences in the interpretation of biosimilarity requirements between legislations at the federal and state level have led to a resurgence of interest in defining precise, statistically testable criteria of prescribability and interchangeability.

In the EU, the procedures of approving a biosimilar and making a statement about its interchangeability are clearly separated. The first is a centralized procedure, i.e. the decision is made at the Community level. The approvals are based on clearly laid-out regulatory requirements [20]. In contrast, to make statements about interchangeability is delegated to the national level. In the 28 member states, different usage patterns have emerged such as substitution in special cases, switching, or restricting the use of biosimilars only to previously untreated patients. The scientific background behind these policies is unclear but setting clear criteria for interchangeability and prescribability are not planned in any of the member states or at the EU level. It is questionable that the situation will ever change because such a discussion would demand a coordinated and comprehensive approach from the EMA and from the competent national authorities. Without clear regulatory criteria, biosimilar producers will not consider sponsoring studies with the goal of demonstrating the interchangeability of their products.

Indeed, there are some initial results and clinical studies that have been actually performed, especially in Europe, between brand-name biologicals and biosimilar products. Such studies have been conducted on products that had been approved by the EMA, including those of granulocyte colony stimulating factors (filgrastim), human recombinant growth hormones (somatotropin) and erythropoietins (epoetin-alfa and epoetin-zeta) [21]. Notably, the switching of erythropoietin products was investigated in several crossover clinical trials [22, 23]. Reviews of these studies could not identify safety risks associated with switching between the biopharmaceutical products [21, 24]. Restricting interchangeability limits the competition which has direct economic consequences. It is acknowledged that interchangeability could be an elusive goal in many cases. This goal is not so elusive when the molecular weight is relatively small and the antibody formation risk is negligible. Hopefully, experience will be gained in time also with comparative studies of biological products. This could lead to formalized regulatory conditions to establish the interchangeability of biological products.
